# Simultaneous text and gesture generation for social robots with small language models

**DOI:** 10.3389/frobt.2025.1581024

**Published:** 2025-05-16

**Authors:** Alessio Galatolo, Katie Winkle

**Affiliations:** ^1^ Department of Information Technology, Uppsala University, Uppsala, Sweden; ^2^ Department of Women and Children’s Health, Uppsala University, Uppsala, Sweden

**Keywords:** social robot, behavior generation, multimodal behavior, deep learning, generative model, interactive behaviors

## Abstract

**Introduction:**

As social robots gain advanced communication capabilities, users increasingly expect coherent verbal and non-verbal behaviours. Recent work has shown that Large Language Models (LLMs) can support autonomous generation of such multimodal behaviours. However, current LLM-based approaches to non-verbal behaviour often involve multi-step reasoning with large, closed-source models-resulting in significant computational overhead and limiting their feasibility in low-resource or privacy-constrained environments.

**Methods:**

To address these limitations, we propose a novel method for simultaneous generation of text and gestures with minimal computational overhead compared to plain text generation. Our system does not produce low-level joint trajectories, but instead predicts high-level communicative intentions, which are mapped to platform-specific expressions. Central to our approach is the introduction of lightweight, robot-specific “gesture heads” derived from the LLM’s architecture, requiring no pose-based datasets and enabling generalisability across platforms.

**Results:**

We evaluate our method on two distinct robot platforms: Furhat (facial expressions) and Pepper (bodily gestures). Experimental results demonstrate that our method maintains behavioural quality while introducing negligible computational and memory overhead. Furthermore, the gesture heads operate in parallel with the language generation component, ensuring scalability and responsiveness even on small or locally deployed models.

**Discussion:**

Our approach supports the use of Small Language Models for multimodal generation, offering an effective alternative to existing high-resource methods. By abstracting gesture generation and eliminating reliance on platform-specific motion data, we enable broader applicability in real-world, low-resource, and privacy-sensitive HRI settings.

## 1 Introduction

The anthropomorphic nature of, e.g., virtual avatars and physically embodied (social) robots simultaneously affords and generates user expectations for multimodal interaction capabilities (Rosén, 2021). Unsurprisingly then, much prior work in Human-Agent/Human-Robot Interaction (HAI/HRI) has been concerned, first, with the design and evaluation of multimodal behaviour, and, second, with the development of methods that can (systematically) support its generation. Interest in developing multimodal robot behaviours is driven by evidence that multimodal communication is important in, and beneficial for, HRI. For example, compared to speech alone, co-verbal hand and arm gestures can boost anthropomorphism, likeability, sense of shared reality and interest in future contact with humanoid robots (Salem et al., 2013; 2011). Similarly, ‘empathetic’ (or not) robot facial expressions, combined with speech, can influence users’ ratings of robot friendship, companionship, alliance, in addition to their own self-validation (Leite et al., 2013). Nevertheless, multimodal behaviour generation remains an open topic of research. A recent review of data-driven communication behaviour for HAI/HRI indicates that the generation of semantically appropriate co-speech behaviour—that is, bodily movements which match agent speech content—remains a challenge (Oralbayeva et al., 2024). The same review also notes that most existent multimodal behaviour generation systems consider each modality in isolation, with simultaneous generation of whole-body gestures, from and with verbal cues, being an open research space of interest in this context.

Going beyond the literature on physical robots reveals a large number of works that aim to generate gestures for, e.g., a generic agent skeleton (Mughal et al., 2024; Habibie et al., 2021; Bhattacharya et al., 2021; Teshima et al., 2022) or virtual face (Habibie et al., 2021) and avatars (Yi et al., 2023), often relying on specialised datasets comprising 3D skeleton or joint movement sequences. While it is sometimes possible to map the gestures present in these datasets onto embodied robot gestures (for example, Yoon et al., 2019 map joint movements to the robot NAO), such mappings are limited in generalisability. Many robotic platforms differ significantly from human morphology, making direct translation of human-like gestures difficult or even undesirable. Furthermore, these systems typically aim to generate low-level pose sequences, which is a different problem space from our focus. Our approach targets the generation of high-level behavioural intents—semantically appropriate communicative actions—rather than detailed motion trajectories. This abstraction allows us to support a broader range of embodiment types, including both facial and bodily gestures, and to remain agnostic to specific kinematic configurations. By operating at a higher level, we prioritise generalisability and reduce reliance on modality-specific or platform-specific datasets, which would constrain the adaptability of the system.

This challenge of creating natural and effective robot gestures becomes even more complex when we consider the rising use of Large Language Models (LLMs) in HRI. With their increased performance and adaptability, their use in HRI studies has skyrocketed—and with it, concerns about their use (Williams et al., 2024). Whilst these concerns span a wide range of topics, we are particularly interested in users’ perceptions and the projected agency onto the robot. Recent studies have revealed that with increased robot communication abilities comes an increase in its expected multimodal behaviour (Kim et al., 2024).

Our research specifically addresses these concerns, with our primary aim being to connect behaviour generation research with LLM integration in Social Robots. To this end, we explore and develop different techniques for end-to-end multimodal robot generation of speech (textual output) and semantically appropriate gestures.

Given that works to date overwhelmingly utilise or rely on closed, high-resource models and/or high availability of data, we specifically set out to investigate the extent we can leverage lower-resource approaches. Here, our motivations are both ethical and pragmatic. On the ethical side, questions have been raised about representativeness and risks of bias associated with existent (large scale) datasets (Bender et al., 2021; Kotek et al., 2023; Omiye et al., 2023; Salinas et al., 2023) (one key reason why some in HRI have explicitly cautioned against their direct deployment on robots Williams et al., 2024). Identifying ways to work with open-source and/or small-data approaches has been associated with increased potential to make LLMs more ethical, and/or, e.g., contextually appropriate/culturally specific (Klein and Ignazio, 2024). On the pragmatic side, we are thinking about the computational resources that might be available on a mobile robot system: it is the case that not all real-world deployments, nor even experimental HRI studies, can be conducted with a constant connection to a remote server that can run large-sized models. For this reason, we push for methods compatible with model sizes that can run “on-device.” In this regard, works on LLMs (Abdin et al., 2024) suggest that 1–3B models would fulfil this constraint if properly optimised (e.g., with 4-bit quantisation), while works in robotics (Nasrat et al., 2025) show that it might be possible to deploy up to 8B models (still with 4-bit quantisation) on more powerful, but still compact, devices such as the NVIDIA Jetson^1^. Finally, we want to draw attention to privacy-constrained settings, such as robots in healthcare, where sensitive data must be handled with great care. Laws such as the General Data Protection Regulation (GDPR) in Europe and the Health Insurance Portability and Accountability Act (HIPAA) in the United States establish requirements for the protection, storage, and sharing of personal health information (Voigt and von dem Bussche, 2017; Act, 1996). These regulations highlight the need for privacy-preserving techniques, especially in environments where the risk of exposing personal information is significant. In such contexts, locally-runnable models are not only a pragmatic choice but also the ethical and socially sustainable one.

Therefore, our research aims to address these challenges by exploring the simultaneous generation of text and gestures using Small Language Models that, at the same time, minimise computational overhead. We focus on developing an approach that can operate efficiently on devices with limited computational capabilities and comply with privacy constraints, thereby expanding the applicability of social robots in various contexts.

**FIGURE 1:**
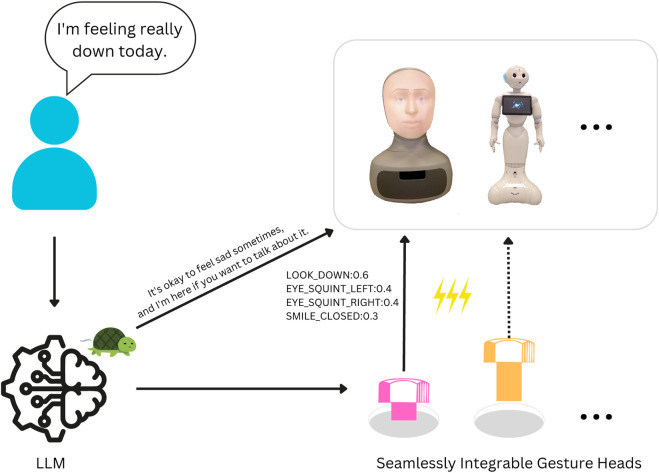
Our method, coupling text and gesture generation through LLMs with minimal overhead, for multiple robot platforms. We highlight the slow (er) text generation process with a turtle and the (much) fast (er) gesture generation with lightning.

We begin our work by analysing and evaluating (Section 3.1, 3.2) common strategies for multimodal (text–gesture) generation (detailed in Section 2.1, Section 2.2) using a variety of language models, highlighting the shortcomings of current methods in both performance and computational needs (Section 3.4). To address these limitations, we introduce the concept of “gesture heads” (Section 2.3), robot-specific modules derived from a given language model that function in parallel with the language modelling head (Figure 1). These modules require minimal computational overhead and no specialised training data. Next, we conduct extensive computational experiments to evaluate the performance of our approach (Section 3.3) on two robot platforms with different non-verbal capabilities: Furhat (facial expressions) and Pepper (body movement). Finally, we demonstrate that our method is effective even when using open-source, Small Language Models, showing the potential for *in-situ* deployment and addressing ethical considerations related to computational sustainability and data privacy.

### 1.1 Related works

In order to position our system and its capabilities with respect to the longstanding interest in and, understanding of/approaches to non-verbal robot behaviours for HRI, we give a short overview of research around non-verbal behaviour in HRI. Then, we identify how LLMs can be used to generate behaviour (single modality) before describing common tactics for LLMs and multimodality outside of HRI. This is done in order to lay the foundations of our method, with which we attempt to overcome current approaches by fusing multimodality in LLMs.

We exclude any works that, whilst addressing gesture/multimodal generation, cannot be easily incorporated into a physical (robotic) body. This is the case for multiple works sitting at the intersection of computer vision and robotics which specifically exploit the high availability of specific data (Mughal et al., 2024; Habibie et al., 2021; Bhattacharya et al., 2021; Yi et al., 2023). This enables specific training routines and architectures, but such data is generally unavailable for most specific robot platforms used in HRI. Moreover, these approaches typically operate at the level of low-level joint trajectories (e.g., 3D skeleton poses), which falls outside the scope of our work. Instead, our focus is on generating high-level communicative intents—abstract representations of behaviour that are intended to be adaptable across different embodiments and interaction contexts. This choice allows for greater generalisability, including support for facial gestures (e.g., as in Furhat) and diverse robot platforms, without being constrained by the availability of specific low-level pose datasets.

#### 1.1.1 Non-verbal behaviour in HRI

Dependent on a particular robot’s embodiment, typical non-verbal behaviours we might expect to (coherently) accompany robot speech may include gesturing and motion (Lim et al., 2011; Bremner et al., 2011; Nguyen et al., 2023), facial expression (Rawal and Stock-Homburg, 2022), proxemics (Mead and Matarić, 2015), paralinguistics (e.g., speaking volume, rate, pitch) (Lim et al., 2011), eye gaze (Admoni and Scassellati, 2017) and touch/haptics (Hoffmann and Krämer, 2021). Numerous studies have indicated the importance of such nonverbal robot behaviour, e.g., for information communication and task performance (Bremner and Leonards, 2015; Bremner et al., 2011; Admoni et al., 2014), for robot persuasiveness (Chidambaram et al., 2012; Nakagawa et al., 2011; Hoffmann and Krämer, 2021; Fischer et al., 2020), and/or for influencing user perceptions of a particular robot platform (Salem et al., 2013; 2011; Leite et al., 2013).

Designing non-verbal behaviours for robots poses significant challenges due to the diversity of embodiments and interaction contexts. Traditional approaches often rely on predefined behaviours or handcrafted rules (Oralbayeva et al., 2024), which can be inflexible and labour-intensive to develop (Breazeal et al., 2005). Examples of hardcoded behaviours can often be seen in studies whose interaction is predefined and constrained (Winkle and Bremner, 2017; Xu et al., 2015). Some works attempt to create more generalisable rule-based systems, for example, Bremner et al. (2009) provide a list of rules for the (hand-scripted) creation of human-like beat gestures, based on a study of chat show hosts. However, the nature of these approaches generally limits their applicability in the context of generating speech-coherent nonverbal behaviour in real-time, although some of them do include, e.g., dynamic responses to users’ own nonverbal behaviours to generate socially appropriate behaviour (Gonsior et al., 2011). Somewhat related here are approaches for shaping or adjusting a pre-defined non-verbal behaviour, e.g., in the context of changing robot affect or personality. For example, Lim et al. (2011)’s DESIRE framework posits the ability to extract generalisable, cross-modality parameters (i.e., speed, intensity, regulation, extent) from one modality (e.g., speech) and apply them to others (e.g., movement) e.g., in order to make it emotionally coherent (Lim et al., 2011).

A common limitation of these methods is their inability to generalise well to dynamic interactions where context-specific behaviours are necessary. Recent advances have explored the use of Machine Learning techniques to generate multimodal behaviours from speech or text input. For instance, gesture generation models have been developed to produce co-speech gestures based on audio features (Ahuja et al., 2020). However, these models often require large amounts of specialised training data, which may not be readily available for all robot platforms or interaction contexts.

In contrast, LLM-based approaches do not generally rely on such extensive specialised datasets. Thanks to their broad, embedded knowledge, they can generate non-verbal behaviours in a more flexible and adaptive manner. By leveraging this understanding, LLMs can infer context-specific actions without explicit rules or predefined behaviours, enabling robots to dynamically respond to varied interaction contexts, thus reducing the need for labour-intensive manual design and extensive data collection processes.

#### 1.1.2 LLMs and behaviour generation

Large Language Models (LLMs), such as GPT-3 (Brown et al., 2020) and GPT-4 (Achiam et al., 2023), have demonstrated remarkable capabilities in generating coherent and contextually appropriate text. Their potential has been recognised in the field of HRI for generating dialogue content and non-verbal behaviours (Parada, 2024).

Mahadevan et al. (2024) develop GenEM and GenEM++ two methods for expressive behaviour generation through in-context learning (Brown et al., 2020) and Chain-of-Thought reasoning (Wei et al., 2022) where GenEM++ improves on the other by incorporating human feedback as the last step of the generation. An interesting insight from their work is that GenEM++ (the feedback-improved model) does not consistently outperform its base version; the authors hypothesise this to be caused by the ‘personal’ nature of feedback it received. Specifically: the feedback loop originated from a single person, but the end results were judged by multiple other people. Further, their method goes through up to four stages of generations (and associated feedback loops) before the final results, greatly hindering the real-time applicability of the method. Liang et al. (2024) develop a method that begins with a similar In-Context Learning-based approach where the interactions are collected to be used for fine-tuning the underlying language model and improve overall ‘teachability’ of future tasks (i.e., a faster adaptation from human feedback). Xu et al. (2024) develop a GesTran, a method that pairs LLMs with auto-encoders to generate full-body gestures from speech.

To the best of our knowledge (and also according to Oralbayeva et al. (2024)) there are no works that jointly produce a text and non-verbal response to a user query, hence the motivation for this work.

Wang et al. (2024) venture *towards* this direction with their method that relies on GPT-4 function calling tools^2^. Here the authors use the language model to produce a “function call” following a high-level goal, e.g., assisting the user in pouring a drink. Amongst the possible functions to be called there is a “speak” function. In this particular work, the interaction is aided by two other modules to translate the low-level input/output into/from high-level ones that can be fed into/produced by the language model. No particular analysis has been conducted to assess if and how non-verbal behaviour relates to the spoken text.

#### 1.1.3 Multimodal LLMs outside HRI

Outside of HRI, there has been significant interest in integrating multiple modalities into LLMs. Common approaches involve augmenting pre-trained LLMs with separate encoders or generators for new modalities, such as vision or audio (Zhang et al., 2024). These methods enable the processing of multimodal input or output but often require substantial computational resources and complex training procedures.

Other approaches, like textual conversion (Song et al., 2023), convert non-text modalities into textual representations that can be processed by LLMs. While this method simplifies integration, it may not fully capture the richness of the original modality. Training LLMs from scratch with multiple modalities is another possibility explored in works like Gato (Reed et al., 2022), but this approach is computationally expensive and impractical for many applications.

## 2 Materials and methods

In this work, we envision the deployment of a robot whose conversational abilities are powered by an LLM for which having paired gestures is highly desirable (as per our Introduction). In such instances, having separate modules for gesture generation may be difficult due to substantial overhead or loss of information between modules.

To achieve this, we explore four different possible paths of gesture generation:

1. In-Context Learning (ICL).
2. Chain-of-Thought (CoT) with stepwise text-gesture derivation.
3. Gesture Heads with In-Context Learning.
4. Gesture Heads alone.

In the first two, we explore the idea of generating gestures as part of the text. Here, each possible gesture is encoded as text (e.g., ‘NECK_TILT’) with its relative parameter (e.g., intensity ∈[−50,50] ). This represents the most naive approach and heavily relies on the world knowledge embedded in the LLM. Further, the LLM will have to be properly prompted with, among other descriptions, a list of all the possible gestures.

While easy to implement, we will show how these methods easily fall apart when decreasing the size of the language model, where most of the generated gestures do not reflect those available to the specific robot platform involved or are not semantically relevant to the generated text.

As a patch to this problem, we introduce the concept of ‘Gesture Heads’, which are small networks added at the end of the language model, taking the last hidden state as input and processing it for gesture generation. The head effectively acts as a classification head, constraining the outputs to the possible parameters available from the given robot platform. Specifically, we will design these heads to be lightweight and easily trainable, taking a negligible amount of parameters compared to the language model itself (≪0.01%) .

### 2.1 In-context learning

We begin our tests by verifying the ability of LLMs on simultaneous text-gesture generation. We prompt the language model based on previous work’s guidelines on robot prompting (Arenas et al., 2023) where the conversation begins with a description of the robot embodiment, followed by its possible movements and a few example interactions for In-Context Learning (ICL) (Brown et al., 2020). In our case, the examples follow the structure defined in Table 1.

**TABLE 1:** Model’s response to “Hello!” when prompted with In-Context Learning.

Hi! [GEST] < set of gestures to compose a greeting > [ \ GEST] Nice to meet you. [GEST] < set of gestures to signify happy response > [ \ GEST]

Here, we make use of two special tokens to delimit the gesture from the normal response and longer responses contain alternating segments of texts and gestures. It is important to note the importance of these delineating tokens, which prove useful not only for the Language Model to understand the right place for the gesture but also to aid the subsequent parsing of the string before its injection into the robot.

Again, we will show this approach is not fit for complex generations due to the compositionality of gestures (i.e., it is not straightforward to combine different gestures to compose another) or the decline in attention with the increase in length of the context.

### 2.2 ICL and Chain-of-thought

Going one step further, we add Chain-of-Thought (CoT) reasoning (Wei et al., 2022) for the gesture generation. Here, we ask the language model to derive the gestures on a step-by-step manner. First, the language model generates a plain text response to the user query, then splits the response into single text segments (one or multiple sentences). For each segment, reasoning is done on what kind of gesture would be appropriate for that segment. Here, the reasoning is unconstrained and done on a high level, which may not reflect the particular robot embodiment. The conversion to a specific robot platform is instead done in the last step of the gesture extraction, where the high-level gesture is converted into robot-specific gestures and parameters. We keep giving examples as before but we change them to reflect this new structure. We show the expected generation in Table 2.

**TABLE 2:** Model’s response to the user query “I”m feeling really down today. when prompted with In-Context Learning and Chain-of-Thought.

# Answer
I’m really sorry to hear that. It’s okay to feel sad sometimes, and I’m here if you want to talk about it
## Text split
I’m really sorry to hear that
### Gesture reasoning
The text conveys empathy and sympathy, typically associated with a gentle downward motion of the brows and a slight frown. This expresses understanding and concern
### Gesture
[GEST] < specific robot gestures > [ \ GEST]
## Text split
It’s okay to feel sad sometimes, and I’m here if you want to talk about it
### Gesture reasoning The tone of this text aims to provide comfort, but it acknowledges the sadness. A soft look downward with subtle squinting of the eyes can show reflective compassion without overwhelming the recipient
### Gesture [GEST] < specific robot gestures > [ \ GEST]

Compared to the previous approach this method is more robust to complex generation and is able to combine multiple gesture in a meaningful way. However, it does introduce *substantial* overhead due to the length in generation.

### 2.3 Gesture head

As introduced previously, one common pitfall of smaller models is the generation or use of non-existent gestures and/or parameters that do not belong to the described robot platform. To patch this problem, we introduce robot-specific gesture heads, these heads act in a similar manner to classification heads and constrain the output to only those available to the robot platform. The head is initialised to mimic the language model’s output when prompted with CoT (Section 2.2), but without its overhead.

The gesture head can be utilised in two ways. One, by pairing it with the textual gesture generations (Table 1), interpreting and patching bad generations, we will refer to this as GH + ICL (Gesture Head with In-Context Learning). In this case, at generation time, the gesture head is applied upon encountering one of the gesture delimiter(s) and matches the textual gestures, possibly non-existing, with the ones available from the platform. We will show how, if provided with proper training, the head can even improve on the quality of the gesture generation.

The second way is to use the head on its own, acting similarly to a separate classifier. Here the head takes the embedding of the textual response and generates appropriate gestures. In this case, at generation time, the gesture head operates in parallel with the language modelling head. When the model generates text, the gesture head processes the hidden states to produce gestures corresponding to the text segments, without requiring special tokens or additional prompting. We will refer to this approach as GH (Gesture Head).

#### 2.3.1 Architecture

**FIGURE 2:**
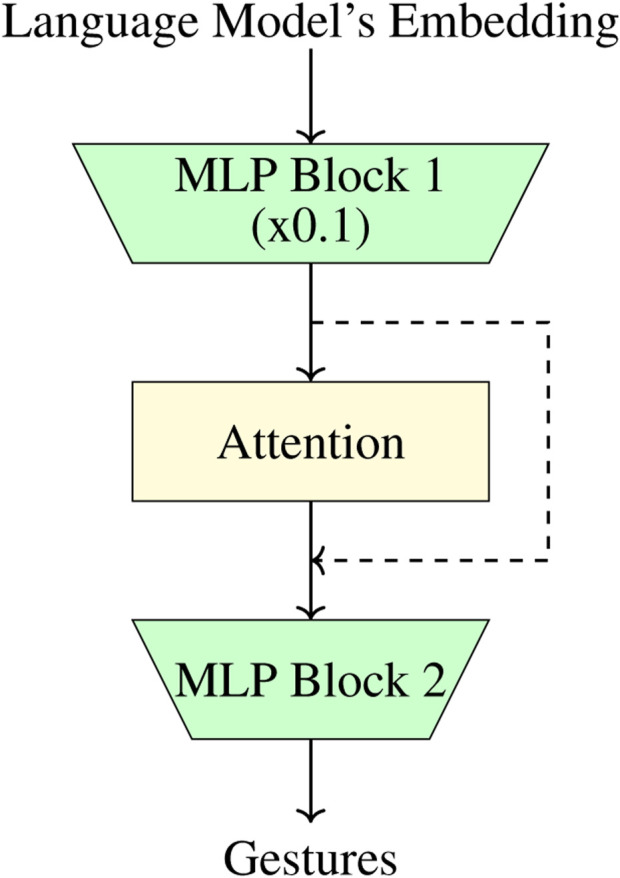
Architecture of the Gesture Head: The input from the language model embedding is passed through a downprojection MLP (reducing the space by a factor of 10), followed by an attention block with residual connections from the first MLP, and finally, another MLP block to output the gestures.

We experiment with different architectures for the gesture head. We begin with a single linear layer, similar to traditional classification heads. Generally, this approach is paired with Supervised Fine-Tuning (SFT) of the whole language model or through the use of Low-Rank Adaptation (LoRA) (Hu et al., 2022) but both of these require extensive resources for training. Given our goal of making the training as lightweight as possible^3^, we exclude any fine-tuning solutions. We instead look at different architectures for the gesture head. After extensive testing, we settle for using two gated Multi-Layer Perceptron (MLP) with a simple Attention block in the middle (shown in Figure 2). For the attention block, we also add residual connections. More in detail, the first MLP block also functions as a down-projection layer, reducing the embedding space by a factor of 10 and reducing the overall parameter count. Our chosen architecture ensures both ease of training and good performance.

#### 2.3.2 Training

Our goal is that the gesture head would mimic the output of its language model when properly prompted (e.g., with CoT) whilst avoiding the computational overhead this introduces *and* fixing the errors due to bad generations. This is achieved through a short training phase, using samples generated by the language model itself.

During training the language model’s weights are kept frozen and we only train the Gesture Head. Here, as we don’t have to back-propagate through the whole model, the training toll is similar to that of training a small MLP. During training, we do a grid search on the learning rate with values in [ 1e−4,1e−5 .

#### 2.3.3 Data augmentation

To improve real-world performance, we also augment the samples with random gestures. For half of the training samples, the correct gestures in the text are replaced with random ones (existing and non-existing) which can also vary in number (can be more or less than the ground truth). The gesture head is then trained to still recognise the correct gesture from the random ones.

For testing, we simulate a real-world situation and adopt an augmentation rate that matches the relative error rate. For example, if a given model (without gesture head) has errors in 17% of its generations, when testing the same model with the gesture head, we introduce random samples in 17% of the samples.

### 2.4 Evaluation samples

In this work, we do not rely on a specific dataset or benchmark because there is no obvious choice that supports the level of generalisability we aim for. While existing datasets could demonstrate the effectiveness of our method, they would not ensure its applicability in real-world conditions.

Nevertheless, to assess the effectiveness of different methods, we have to derive evaluation examples. To maintain our original premise, we design this process so that it does *not* require human expertise or intervention. Our evaluation examples consist of user queries paired with corresponding gestures. These examples are synthetically generated using a relatively large LLM, prompted with Chain-of-Thought (CoT) reasoning to generate gestures step-by-step. Based on prior work (Mahadevan et al., 2024; Xu et al., 2024; Liang et al., 2024), we expect this approach to produce reasonably accurate gestures. These are not intended to be optimal gestures but serve as a reasonable baseline for comparing different methods and models.

This approach remains independent of human expertise, except for defining a set of possible gesture parameters for the model to generate. For the base interactions, we use the SODA dataset (Kim et al., 2022), a publicly available collection of everyday textual interactions that serves as a general-purpose dataset representative of common conversational scenarios. Crucially, this dataset is not specialised for any particular domain or robot platform, ensuring that our method remains broadly applicable without relying on domain-specific training data. This generality supports privacy and inclusivity while also allowing easy substitution with other general conversational datasets.

Notably, while this evaluation data is primarily used for assessing performance, it can also be leveraged to train gesture heads, as defined earlier. This effectively simulates a knowledge distillation setting (Hinton et al., 2015), where outputs from a larger model improve the performance of the smaller gesture head. Importantly, this does not contradict our emphasis on using smaller models for efficiency, privacy, and ethical considerations—–these benefits apply once the gesture heads are trained. The use of larger models at this stage is simply a means of bootstrapping better gesture representations without requiring costly human annotations.

### 2.5 Metrics

To evaluate and compare the different methods in this work, we employ a set of metrics that account for both performance and computational requirements.

For computational efficiency, we consider two factors: generation time (wall-clock time) and memory usage (VRAM consumption). In both cases, we focus on the additional cost introduced by multimodal generation compared to plain text generation. Rather than reporting absolute values, we express results as relative overheads—for instance, if a multimodal generation method takes twice as long as text-only generation, we report it as ×2 . This approach makes our evaluation inherently hardware-agnostic.

For performance evaluation, we use four metrics: Accuracy, F1 score, Overlap, and Error rate. The Error rate quantifies mistakes in gesture generation when represented as text (e.g., using methods described in Section 2.1 and Section 2.2). Errors occur when a model generates an invalid or misspelt gesture name, making the gesture unprocessable. The Error rate is defined as the proportion of erroneous gesture segments over the total number of segments.

Accuracy and F1 score are standard classification metrics and are applicable only in the context of gesture heads (Section 2.3), where gestures are predicted similarly to class labels. However, for text-based approaches (e.g., ICL and CoT), these metrics cannot be computed directly. Instead, we introduce Overlap, which measures how many generated gestures match the ground truth. Given a gesture segment, let $G_{g}$ be the set of generated gestures and $G_{t}$ the ground truth set. The Overlap metric is defined as:

$$\text{Overlap}=\frac{\left|G_{g}\cap G_{t}\right|}{\left|G_{t}\right|}$$

In our evaluation, we directly compare Overlap with Accuracy, as both quantify the rate of correctly predicted gestures.

## 3 Results

To showcase our method, we select LLaMA 3.1 (Dubey et al., 2024) as the family of models that we will use. In particular, we use the 70B version as an example of LLM, and the 8B version as an example of a language model runnable on consumer hardware. Finally, we also test the 1B and 3B versions of LLaMA 3.2 (Meta AI, 2024) as an example of locally-runnable (on-device) SLMs. Whilst, to the best of our knowledge, no robot commonly available to HRI researchers is capable of using on-device language models, this is likely to change in the upcoming years thanks to the progress in processing units. With this in mind, the ability to deploy robots in the wild that are capable of autonomous text processing and generation becomes increasingly feasible, leading to an extension of current robot application contexts.

With regards to the robot platform, we have worked to make this method as platform-agnostic as possible. To showcase its flexibility and effectiveness we work here with the platforms 1) Furhat (Al Moubayed et al., 2012) and 2) Pepper (Pandey and Gelin, 2018) as 1) an example of a robot capable of complex facial expressions and 2) a robot capable of various hand gestures and arm movements.^4^

### 3.1 ICL performance

As we introduced in the methodology section, we begin our experiments by testing whether language models are capable of generating textual gestures alongside their normal replies with just In-Context Learning. By manually analysing the generations, we notice that, as the size of the language model decreases, its tendency to make errors in generations increases. We show in Table 3 the error rate in generation when varying on the model size and robot platform. As one might expect, the biggest model is the one with the lowest error rate which gradually increases with the decrease of model size.

**TABLE 3:** Performance of gesture generation when using ICL and generating gestures alongside text. Error rate refers to mistakes in gesture parameters (e.g., non-existing gesture name, impossible intensity, etc.) while overlap refers to the overlap with some ground-truth gestures.

Model Size	Error rate ↓		Overlap ↑	
	Furhat	Pepper	Furhat	Pepper
70B	**3%**	**2%**	30.8%	**25.1%**
8B	27%	62.2%	**32%**	20.8%
3B	29.3%	35.7%	20%	19.2%
1B	42.5%	55.2%	13.7%	12.8%

The bold values represents the best value in each column.

In addition to error rate, we also consider an additional accuracy metric. Error rates only give an idea of the usability of a certain method/model but do not give any information on their quality. For this, we collect a set of “ground-truth” gestures through the use of CoT reasoning and our biggest model. In this case, the accuracy assesses the overlap between the model’s own generated gestures and the ground truth as defined above. Of course, language models’ generations are not deterministic so a perfect score is impossible for any model, nevertheless, we consider this as the main metric of generation quality.

### 3.2 CoT performance

We report in Table 4 the performance of various models on the same metric as Table 3 but when using Chain-of-Thought reasoning. Looking at the accuracy, there is a general improvement (although less consistent across models compared to the ICL-only case), indicating CoT’s effectiveness in generating better gestures. The error rate is variable, increasing or decreasing depending both on the model and robot platform. This may indicate the complexity in generating a more structured output, where the model first needs to split its response into single text segments, then reason on that segment and finally generate the gesture.

**TABLE 4:** Performance of gesture generation when using ICL and CoT and generating gestures alongside text. Accuracy for the 70B model is not reported as it is taken as ground truth.

Model Size	Error rate ↓		Accuracy ↑	
	Furhat	Pepper	Furhat	Pepper
70B	**12%**	**6%**	-	-
8B	57.8%	49.3%	31.9%	24.7%
3B	35%	34.14%	**33.64%**	**28.65%**
1B	60.2%	41.5%	18.6%	16.5%

The bold values represents the best value in each column.

### 3.3 Gesture head

From the previous analysis, we know that prompt engineering is not enough to solve the task we have at hand. Where, e.g., in the case of Pepper, almost one in two gesture generations (across models, excluding the 70B version) contain errors, essentially infacilitating their real-world deployment. To address this, we train a gesture head to aid in the gesture generation process.

We report in Table 5 the test accuracy and F1 metric. Note how the accuracy in this Table can be directly compared to that in Tables 3, 4 as they are both assessing the number of correct samples over the same dataset. Based on this, we can see a great increase in performance, although varying on the robot platform.

**TABLE 5:** Accuracy and F1 metric of the gesture head on the test set across different model sizes.

Model Size	Accuracy ↑		**F1** ↑	
	Furhat	Pepper	Furhat	Pepper
70B	32.1%	69.1%	30%	68.9%
8B	**50%**	**71.2%**	**49%**	**71%**
3B	45.8%	70.1%	44.8%	69.9%
1B	45.0%	66%	44%	65.7%

The bold values represents the best value in each column.

### 3.4 Overhead analysis

As mentioned in the beginning of our methodology section, one of our goals is that of pairing textual response with gestures without introducing additional overhead, or at least minimising it. For this reason, it is important to minimise the length of the input sequence as the computational resources needed scale quadratically with its length (Vaswani et al., 2017). In Table 6 we show the overhead introduced by each method. We express the overhead as a multiplier of the time or memory needed for the generation compared with just generating a plain text response. Among all the methods we tested, the gesture head is the only one with almost no overhead, while Chain-of-Thought reasoning introduces the biggest one.

**TABLE 6:** Gesture generation overhead for In-Context Learning (ICL), Chain-of-Though (CoT), Gesture Head (GH), GH and ICL. The overhead is expressed as a multiplier of the time or memory needed for the generation compared with just generating plain text.

Method	Overhead ↓	
	Time	Space
ICL	× 3.3	× 5.8
CoT	× 6.6	× 12.2
Only GH	× **1.02**	× **1.5**
GH and ICL	× 3.3	× 8

The bold values represents the best value in each column.

### 3.5 On-robot demonstration: Proof of concept

To demonstrate proof of concept, we implemented our method on the two robotic platforms: Furhat and Pepper. Reviewing the output behaviours, we observed improvements in gesture accuracy and coherence, particularly with the gesture head correction mechanism. Whilst full human user evaluation is required to comment concretely, we observed that the generated gestures aligned well with the intended communicative goals of the robot in several common HRI tasks, such as greeting and storytelling. These demonstrations show the robots producing appropriate non-verbal behaviours synchronised with their speech, enhancing the naturalness of the interaction.

**FIGURE 3:**
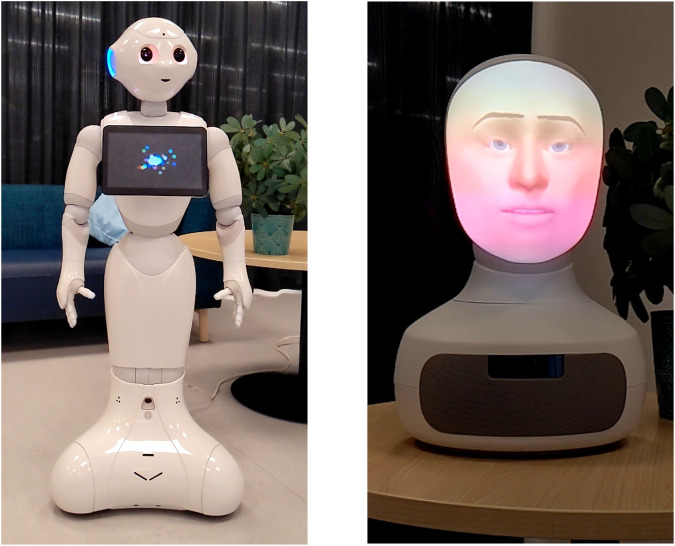
Snapshots of videos illustrating our proposed method when integrated into a robot.

The performance, particularly with the Furhat robot, highlighted the need for further fine-tuning (as also suggested by our qualitative metrics). In particular, we chose to manifest the gestures alongside the speech and while this works well for Pepper, it creates weird situations in Furhat where, e.g., it is trying to smile while also talking. Nevertheless, our tests were overall promising and we are hence optimistic about the potential of this method. Snapshots of this interaction are shown in Figure 3 and we include a video of the complete interaction in the Supplementary Material.

### 3.6 Edge devices performance

To show how our method may behave on edge devices, we have deployed it on an NVIDIA Jetson Orin Nano (8 GB). Similar to other works in robotics Nasrat et al. (2025), we report in Table 7 the number of tokens/s generated alongside varying model sizes and precision. Here, we notice that our method’s speed is comparable to that of the plain LLM across various model sizes and quantisations.

**TABLE 7:** Edge device speed (tokens/s ↑ ) of our method vs. plain LLM for different model sizes and precisions. ‘-’ indicates that a particular model-precision combination did not fit in the device.

Model Size	Text only			Ours		
	bf16	int8	int4	bf16	int8	int4
1B	15.42	4.48	10.58	15.24	4.47	10.49
3B	-	2.67	6.82	-	2.65	6.80
8B	-	-	5.54	-	-	5.47

Importantly, this finding highlights the efficiency of our approach in resource-constrained environments. The differences between the plain LLM and our method are negligible—never exceeding 0.1 tokens/s—across all tested configurations, including int4 quantised 8B models, which are among the most compute-efficient. This validates our design goal: achieving high-level multimodal generation without compromising inference speed. For example, in the 1B and 3B int4 models, the gap between the plain LLM and our system is less than 0.03 tokens/s, which is effectively imperceptible during interaction. Notably, even the largest model tested (8B, int4) runs above 5 tokens/s, which supports real-time dialogue and gesture production on-device.

These results reinforce the claim that our system introduces minimal overhead while enabling rich multimodal behaviour, thus making it well-suited for deployment in social robots operating in low-power or privacy-sensitive scenarios. Given that many state-of-the-art multimodal systems require either large-scale servers or multi-stage processing pipelines, our approach offers a practical alternative that retains performance without sacrificing responsiveness or adaptability.

### 3.7 Reflections

Our experiments demonstrate that the gesture head effectively enables simultaneous text and gesture generation with minimal computational overhead. The approach improves the performance of smaller models, making them suitable for deployment in resource-constrained environments.

Our results also highlight the limitations of relying solely on ICL or CoT, particularly for smaller models where error rates and accuracy significantly hinder their real-world use. Our gesture head, obtained with minimal resource requirements, addresses these limitations by providing a specialised module that ensures compatibility with the robot’s embodiment and improves the final accuracy.

## 4 Discussion

In this paper, we set out to achieve simultaneous generation of text and robot-specific gestures through LLMs, with a particular focus on producing high-level communicative behaviours rather than low-level pose sequences. Our system is not designed to operate at the level of precise joint trajectories, but instead to model and predict appropriate behavioural intents in interaction contexts. This design decision is central to the generality and modularity of our approach.

To support this goal, we specifically choose to 1) use open models and 2) avoid the collection of specialised datasets, based on considerations ranging from ethical and privacy-related to pragmatic. Instead, we employ a generic, platform-agnostic dataset that does not constrain gesture representations to a specific robot or modality. This allows us to leverage the embedded world knowledge of LLMs and generalise to a wide range of platforms, including those capable of facial expressions (e.g., Furhat), without requiring dataset-specific adaptation.

We begin our exploration by taking inspiration from previous works and experimenting with different prompting techniques, tested across various sizes of language models. Our results show how smaller models exhibit a significant number of errors in their gesture generation process. This is vastly mitigated in their larger counterparts which, however, still present unsatisfactory results. Further, our experiments on the computational requirements show how these techniques add a significant overhead to the generation—up to ×6.6 the time and ×12.2 the memory.

To patch the errors made by the language model, we introduce the concept of gesture heads, a small network added at the end of the language model to constrain and improve the gesture generation process. The gesture heads are robot-specific but require minimal training (a few minutes on consumer-grade hardware) and function in parallel with the language modelling head. Our experiments demonstrate that the gesture head significantly improves the performance of smaller models, reducing error rates and enhancing accuracy without introducing substantial computational overhead. This makes it feasible to deploy our method on robots with limited computational resources, expanding the applicability of social robots in various contexts.

Finally, we test our method on Furhat and Pepper, two robot platforms exhibiting complementary non-verbal behaviours. While we do not conduct any human evaluation, initial tests show promising results and demonstrate the effectiveness of our method with improved speed and gesture coherence, especially thanks to the gesture head mechanism.

### 4.1 Limitations and future work

While our method addresses several challenges in multimodal behaviour generation, there are limitations to consider. Crucially, our approach is not intended for low-level gesture reproduction, such as precise joint control or pose sequence replication. As such, methods and datasets designed for pose-level evaluation (e.g., 3D skeleton datasets) are not aligned with our goals and would not adequately capture the communicative behaviours we seek to model.

Our approach focuses on gestures derived from text input. Incorporating additional modalities, such as audio features (e.g., prosody, intonation), could enhance the naturalness of the generated behaviours but would require extending the model to process multimodal inputs. Also, our evaluation relies on the overlap with gestures generated by our largest model, which we take as a proxy ground truth. While this aligns with our focus on high-level behavioural intent, future human user studies would provide deeper insights into the perceived naturalness and appropriateness of the generated behaviours.

Another promising research path is that of Adaptive Learning, implementing online learning mechanisms to adapt the gesture head based on user feedback during interactions. Compared to other works that adapt or fine-tune the entire language model, our method only involves small gesture heads. This is likely to reduce the number of samples and computational resources required, enabling more responsive, per-interaction adaptation.

## Data Availability

The original contributions presented in the study are included in the article/Supplementary Material, further inquiries can be directed to the corresponding author.

## Footnotes

1
https://www.nvidia.com/en-us/autonomous-machines/embedded-systems/

2
https://platform.openai.com/docs/assistants/tools/function-calling

3 Our final solution only requires 5 min of training on consumer-grade hardware with a similar memory footprint as that of inference-time.

4 We include in the Supplementary Material all the code for our method, the deployment in these specific robot platforms and instructions on how to expand it to other robot platforms.
